# Clinicopathogenomic analysis of *PI3K/AKT/PTEN*-altered luminal metastatic breast cancer in Japan

**DOI:** 10.1007/s12282-024-01639-6

**Published:** 2024-10-28

**Authors:** Hiroshi Tada, Minoru Miyashita, Narumi Harada-Shoji, Akiko Ebata, Miku Sato, Tokiwa Motonari, Mika Yanagaki, Tomomi Kon, Aru Sakamoto, Takanori Ishida

**Affiliations:** Department of Breast and Endocrine Surgical Oncology, Graduate School of Medicine, Tohoku University, 1-1 Seiryo-machi, Aoba-ku, Sendai, Miyagi 980-8574 Japan

**Keywords:** *PI3K/AKT/PTEN* alterations, Metastatic recurrent breast cancer, AKT pathway mutations, Comprehensive genomic profiling, AKT inhibitors

## Abstract

This rapid communication highlights the correlation between protein kinase B alpha (*AKT1*)-phosphatidylinositol-4,5-bisphosphate 3-kinase catalytic subunit alpha (*PIK3CA*)- phosphatase and tensin homolog (*PTEN*) alterations and clinicopathological factors in Japanese patients with metastatic recurrent breast cancer (mBC). This study analyzed 1967 patients with luminal-type breast cancer who underwent cancer gene panel testing. The results demonstrated that AKT pathway alterations, including *PI3K/AKT/PTEN*, occurred in 1038 (52.8%) cases. Patients with AKT pathway mutations were older (p = 0.002) and had a higher rate of invasive lobular carcinoma (ILC) histology (p = 0.001), progesterone receptor (PgR) positivity (p = 0.006), and bone metastases (p = 0.001), and a lower rate of germline BRCA2 (p < 0.001). Comprehensive genomic profile results demonstrated a higher tumor mutational burden (TMB) (< 0.001) and lower tumor BRCA1/2 expression (< 0.001) in patients with mutations in the AKT pathway. These results are crucial for characterizing candidates for AKT pathway-targeted molecular therapies and conceptualizing optimal treatment strategies. *Clinical trial registration: *This study is an observational study and is therefore not registered with the clinical trials registration.

**Supplementary Information:**

The online version contains supplementary material available at 10.1007/s12282-024-01639-6.

## Introduction

Breast cancer is a common fatal malignancy in women worldwide [1]. Hormone receptor-positive, human epidermal growth factor receptor 2 (HER2)-negative luminal types constitute approximately 70% of all breast cancers [2]. Despite advancements in treating metastatic recurrence in luminal types using cyclin-dependent kinase (CDK)-4/6 inhibitors, disease progression remains a major clinical challenge for most patients [3].

In recent years, genetic approaches for breast cancer have been promoted, and precision medicine using molecularly targeted therapeutics has been introduced [4]. The protein kinase B-phosphatidylinositol-4,5-bisphosphate 3-kinase catalytic subunit alpha-phosphatase and tensin homolog (*PI3K/AKT/PTEN*) pathway, a tumor molecular signaling pathway in breast cancer, is a major signaling pathway in various types of cancer that contributes to tumor development and progression [5]. Activation of this pathway begins with ligand binding to the corresponding receptor tyrosine kinase, phosphoinositide 3-kinase (PI3K). The regulatory subunit of PI3K is phosphorylated, and its catalytic subunit p110 is activated. Activated PI3K generates the lipid second messenger phosphatidylinositol 3, 4, 5-triphosphate (PIP3) and downstream effectors, such as AKT and mammalian target of rapamycin protein (mTOR), thereby activating *PTEN*. In contrast, it inhibits PI3K function by dephosphorylating PIP 3, making it a major natural inhibitor of the PI3K-AKT-mTOR pathway [6]. Over-activation of this pathway occurs in approximately half of luminal-type breast cancers owing to activating mutations in *PIK3CA* and *AKT1* and inactivating alterations in *PTEN* [7]. Abnormalities in this pathway are associated with resistance to endocrine therapy and approaches to inhibit this pathway have been attempted. The mTOR inhibitor everolimus was combined with exemestane in the Breast Cancer Oral Everolimus Study 2 (BOLERO-2) trial and exhibited greater efficacy than that of endocrine therapy alone, although no predictive biomarkers currently exist [8]. The PI3K alpha-selective inhibitor alpelisib has been used in combination with fulvestrant in *PIK3CA* mutant tumors and demonstrated greater efficacy than endocrine therapy alone [9, 10]. However, alpelisib is not approval in Japan because of the high incidence of adverse events. The AKT inhibitor capivasertib in combination with fulvestrant resulted in significantly longer progression-free survival than fulvestrant alone in patients with hormone receptor-positive advanced breast cancer exhibiting disease progression during or after previous aromatase inhibitor therapy, with or without a CDK4/6 inhibitor [11]. Overall, 708 patients were randomized in this trial and 289 (40.8%) had an altered AKT pathway; in the AKT pathway-mutated population, median progression-free survival was 7.3 months in the capivasertib plus fulvestrant arm, compared with 3.1 months in the placebo plus fulvestrant arm. The hazard ratio was 0.50 (95% CI 0.38–0.65, P < 0.001). Based on these results, capivasertib and a companion diagnostic device were approved by the Food and Drug Administration (FDA) in November 2023. Additionally, it was covered by insurance in Japan in May 2024, and the detection of *PIK3CA*, *AKT1*, and *PTEN* alterations by FoundationOne CDx is a companion diagnosis for the use of capivasertib in HR-positive HER2-negative advanced breast cancer with *PIK3CA*, *AKT1*, or *PTEN* alterations.

In Japan, clinical genomic data from over 60,000 patients who underwent cancer genome profiling have been collected from a national central database, the Cancer Genome Information Center (C-CAT) [12]. These extensive data include details on histology, genetic mutations, and therapeutic information including metastatic recurrent breast cancer (mBC) in Japan. Therefore, this study aimed to assess the clinicopathological correlation between the frequency of *PIK3CA*, *AKT1*, and *PTEN* alterations in luminal-type mBC patients undergoing cancer gene panel testing in Japan, using data from the Center for Cancer Genome Information and Advanced Therapy (C-CAT) database.

## Materials and methods

Comprehensive genomic profiling (CGP) can only be used in patients with advanced solid tumors who have completed standard chemotherapy or who lack appropriate standard chemotherapy options. The CGP results were explained to the patient by their physician after review by an Expert Panel (EP). Clinical and genetic mutation data obtained through CGP were provided to the C-CAT with patient consent. In Japan, two tissue-based CGP tests, Foundation One® CDx (F1CDx) and OncoGuide™ NCC Oncopanel (NCC Oncopanel), became reimbursable under the National Health Insurance Scheme administered by the Ministry of Health, Labour, and Welfare in Japan in June 2019. Additionally, liquid-based Foundation One® Liquid CDx was covered in 2021. Moreover, the liquid-based Guardant360 and tissue-based GenMine Top Panel were covered in June 2023. The CGP results and clinical data from nearly all patients who underwent CGP testing were collected from the C-CAT [13]. The study used data from patients with breast cancer who consented to both data registration with the C-CAT and secondary use of their data.

From June 2019 to February 2024, C-CAT included 4084 mBC cases with annotated genomic profiles Among these, 1967 were classified as luminal-type breast cancer, characterized by estrogen receptor (ER) positive and/or progesterone receptor (PgR) positive, and HER2 negative (C-CAT Ver. 20240219). A flow diagram is shown in Supplementary Fig. 1.

Clinical data collected included patient age, sex, histological subtype, CGP testing type, post-CGP treatment, date of diagnosis and last observation, and survival status at the last observation. Data on annotated genetic alterations were collected using the C-CAT-provided OncoKB, ClinVar, and Cancer Knowledge databases. Gene alterations in the pathogenic or possibly pathogenic AKT pathways-altered (*PIK3CA*, *AKT1*, or *PTEN*) were extracted from this database.

The companion diagnostic genes for capivasertib were based on CAPItello-291, with only E17K for *AKT1* and 19 short variants of *PIK3CA* (R88Q, N345K, C420R, E542K, E545A, E545D, E545Q, E545K, E545G, Q546E, Q546K, Q546R, Q546P, M1043V, M1043I, H1047Y, H1047R, H1047L, and G1049R). *PTEN* included 13 short variants (C124R, C124S, G129E, G129V, G129R, R130Q, R130G, R130L, R130P, C136R, C136Y, S170R, and R173C) and any nonsense, frameshift, or splice site alterations. *PTEN* rearrangement was assumed to be a homozygous deletion of one or more exons, or any event that disrupts protein function, such as intragenic events (duplications of only part of the gene, deletions, inversions, and translocations) regardless of the transcript. Capivasertib companion diagnostic (CDx) genes were defined accordingly. Alterations of *AKT1*, *PIK3CA*, and *PTEN* other than the capivasertib companion gene were defined as non-CAPI CDx.

High tumor mutational burden (TMB) was defined as having at least 10 mutations per megabase (Mb) in the tissue-based panel and at least 14 in the liquid-based panel.

Cases involved individuals who had previously undergone germline BRCA1/2 testing with negative results, but the panel test demonstrated somatic BRCA1/2 gene alterations.

Based on the mutation data, the gene mutation profile was visualized using the OncoPrinter platform on cbiportal.org (accessed on June 11, 2024).

Statistical analysis was performed using R and its command input platform ezR [14]. Categorical variables were compared using the χ 2 test or Fisher’s direct probability test.

The study was approved by the Ethics Committee of Tohoku University Graduate School of Medicine (permission no. 2021-1-681) and the C-CAT Data Access Review Committee (CDU2022-008E03).

## Results

In summary, of the 1967 patients, 1038 (52.8%) had *PI3K/AKT/PTEN* gene alterations. Table 1 presents the clinical characteristics of the AKT pathway-altered and unaltered groups, in which the mutation was absent. The age distribution was significantly higher in the AKT pathway-altered group (P = 0.002). The histological type demonstrated a higher proportion of invasive lobular carcinoma (ILC) in the AKT pathway-altered group (8.8 vs. 5.1% [p = 0.001 when considering ILC and non-ILC]). Immunohistological testing revealed higher proportion of PgR positivity (p = 0.006) in the AKT pathway-altered group. Among the cases with germline BRCA1/2 testing, the AKT pathway-altered group exhibited a lower proportion of BRCA1 variants (p = 0.051) and significantly fewer BRCA2 variants (p < 0.001). There was no difference in the number of metastatic organs at the time of the panel examination. At the metastatic site, there was no difference in the presence or absence of brain, lung, lymph node, or liver metastasis; however, only the proportion of bone metastasis was significantly higher in the AKT pathway-altered group (p = 0.007). The proportion of bone metastases alone did not differ significantly between the two groups (p = 0.338). The median number of drug therapy regimens received before test submission was similar between the AKT pathway-altered and unaltered groups, with a median of six (1–17) and six (1–20) regimens, respectively. Based on the cancer gene panel test type, the AKT pathway-altered group had a higher proportion of FoundationOne CDx than that in the unaltered group (70.6 vs. 62.2%). Overall, the proportion of tissue-based panels was significantly higher than that of the liquid-based panels (p = 0.011).

**Table 1:** Clinicopathological features

Factor	Group	AKT pathway-altered	Unaltered	p.value
n		1038	929	
Age (mean, range)		56.96 (10–35)	55.44 (10–93)	0.002
Age(%)	<50	257 (24.8)	294 (31.6)	0.001
	≧50	781 (75.2)	635 (68.4)	
Gender (%)	Woman	1034 (99.6)	917 (98.7)	0.041
	Man	4 (0.4)	12 (1.3)	
PS (%)	0	610 (58.8)	552 (59.4)	0.685
	1	376 (36.2)	338 (36.4)	
	2	33 (3.2)	26 (2.8)	
	3	6 (0.6)	2 (0.2)	
	4	2 (0.2)	0 (0.0)	
	Unknown	11 (1.1)	11 (1.2)	
Histological type (%)	Invasive Ductal Carcinoma	834 (80.3)	744 (80.1)	<0.001
	Invasive Lobular Carcinoma	91 (8.8)	47 (5.1)	
	Other type	113 (10.9)	138 (14.9)	
ER (%)	Positive	1026 ( 98.8)	912 (98.2)	0.262
	Negative	12 (1.2)	17 (1.8)	
PgR (%)	Positive	767 (73.9)	627 (67.5)	0.006
	Negative	260 (25.0)	296 (31.9)	
	Undecidable	5 (0.5)	3 (0.3)	
	Unknown or uninspected	6 (0.6)	3 (0.3)	
HER2 IHC (%)	Boundary area (score 2+)	170 (16.4)	149 (16.0)	0.15
	Negative (score 1+)	370 (35.6)	375 (40.4)	
	Negative (score 0)	483 (46.5)	390 (42.0)	
	Unknown or uninspected	15 (1.4)	15 (1.6)	
gBRCA1 (%)	Negative	680 (98.7)	598 (97.1)	0.051
	Positive	9 (1.3)	18 (2.9)	
gBRCA2 (%)	Negative	658 (95.4)	528 (85.6)	<0.001
	Positive	32 (4.6)	89 (14.4)	

Factor	Group	AKT pathway-altered	Unaltered	p.value
n		1038	929	
Number of metastatic sites (%)	1	214 (20.6)	189 (20.3)	0.793
	≧2	813 (78.3)	727 (78.3)	
	N/A	11 (1.1)	13 (1.4)	
Bone metastasis (%)	Yes	662 (63.8)	537 (57.8)	0.007
	No	376 (36.2)	392 (42.2)	
Bone metastasis only (%)	Yes	54 (5.2)	39 (4.2)	0.338
	No	984 (94.8)	890 (95.8)	
Lymphnode metastasis (%)	Yes	445 (42.9)	415 (44.7)	0.439
	No	593 (57.1)	514 (55.3)	
Lymph metastasis node only (%)	Yes	27 (2.6)	31 (3.3)	0.353
	No	1011 (97.4)	898 (96.7)	
Brain metastatsis (%)	Yes	86 (8.3)	65 (7.0)	0.309
	No	952 (91.7)	864 (93.0)	
Brain metastasis only (%)	Yes	0 (0.0)	1 (0.1)	0.472
	No	1038 (100.0)	928 (99.9)	
Liver metastasis (%)	Yes	638 (61.5)	557 (60.0)	0.517
	No	400 (38.5)	372 (40.0)	
Liver metastasis only (%)	Yes	88 (8.5)	73 (7.9)	0.508
	No	950 (91.5)	856 (92.1)	
Lung metastasis (%)	Yes	329 (31.7)	308 (33.2)	0.5
	No	709 (68.3)	621 (66.8)	
Lung metastasis only (%)	Yes	21 (2.0)	21 (2.3)	0.756
	No	1017 (98.0)	908 (97.7)	
No.of preEP regimen (median, range)		6 [1–17]	6 [1–20]	0.764
No.of preEP regimen (%)	1	48 (4.6)	40 (4.3)	0.931
	2	35 (3.4)	29 (3.1)	
	3	72 (6.9)	57 (6.1)	
	4	106 (10.2)	106 (11.4)	
	≧5	753 (72.5)	675 (72.7)	
	N/A	24 (2.3)	22 (2.4)	
Panel name (%)	FoundationOne CDx	733 (70.6)	578 (62.2)	0.001
	FoundationOne Liquid CDx	205 (19.7)	224 (24.1)	
	NCC OncoPanel	94 (9.1)	118 (12.7)	
	Guardant360 CDx	5 (0.5)	9 (1.0)	
	GenMineTOP	1 (0.1)	0 (0.0)	
Panel.type (%)	Tissue based panel	828 (79.8)	696 (74.9)	0.011
	Liquid based panel	210 (20.2)	233 (25.1)	
Specific.collection.site (%)	Breast	407 (39.2)	355 (38.2)	NA
	Liver	137 (13.2)	138 (14.9)	
	Lymph nodes	74 (7.1)	75 (8.1)	
	Skin	69 (6.6)	61 (6.6)	
	Lung	34 (3.3)	16 (1.7)	
	Bone	14 (1.3)	3 (0.3)	
	Brain	12 (1.2)	4 (0.4)	
	Peripheral blood	210 (20.2)	233 (25.1)	
	Others	81 (7.8)	44 (4.7)	

Comparison between AKT pathway-altered and -unaltered groups

*PS* performance status, *IHC* immunohistochemistry, *EP* expert panel

**Fig. 1:**
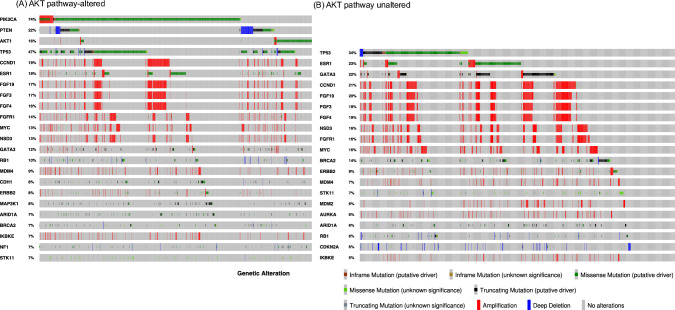
Alteration status using Oncoprint plot for luminal type metastatic recurrent breast cancer (mBC) in Japan. **A** Oncoprint plot of 1038 patients in the AKT pathway-altered group. **B** Oncoprint plot of the unaltered group, in which no alteration was observed in the AKT pathway. Top genes are extracted from each visualized gene

The genomic landscape of the AKT pathway-altered and -unaltered groups is illustrated in Fig. 1, in which *PIK3CA* alterations were the most common (74%), followed by *PTEN* (22%) and *AKT1* (18%). *AKT1* was the most commonly expressed protein. In addition to these three mutations, the most common coexisting mutations in the AKT pathway-altered group were tumor protein 53 (TP53, 47%), cyclin D1 (CCND1, 19%), ESR1 (19%), fibroblast growth factor 19 (FGF19, 17%), FGF3 (17%), FGF4 (16%), and fibroblast growth factor receptor (FGFR1, 14%). The unaltered group exhibited a similar trend. However, TP53 was observed in 47% of the AKT pathway-altered group, which was higher than that observed in the unaltered group (34%). The frequency of Erb-B2 receptor tyrosine kinase 2 (*ERBB2*) alterations, including amplification, was 8 and 9% in the AKT pathway-altered and unaltered groups, respectively. The percentage of BRCA2 mutations was 7 and 14% in the AKT pathway-altered and unaltered groups, respectively. Detailed frequencies of each alteration are listed in Supplementary Table 1.

**Fig. 2:**
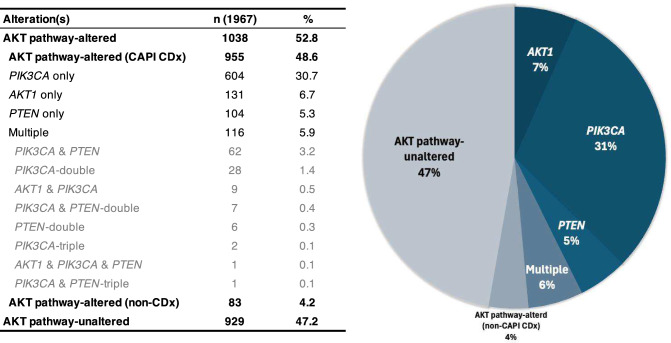
Distribution of AKT pathway alterations. The distribution of mutations per case is illustrated; multiple represents cases in the CAPI companion diagnostic (CDx) group, in which multiple AKT pathway gene mutations are observed in the same case. CAPI CDx indicates companion genes of capivasertib

Figure 2 illustrates the proportion of alterations in the AKT pathway and their breakdown. The overall proportion of AKT pathway alterations was 52.8% (1038 cases), of which 48.6% (955 cases) were in the CAPI CDx group, 4.2% (83 cases) in the non-CAPI CDx group, and 47.2% (929 cases) in the no mutation group. In CAPI CDx group, 30.7% had *PIK3CA*, 6.7% had *AKT1*, and 5.3% had *PTEN*. More than one of the three genes was present in 5.9% of the cases, with *PIK3CA* and *PTEN* being the most common (62 cases, 3.2%), followed by a double alteration of *PIK3CA* in 28 cases (1.4%). Four patients (0.3%) had co-alterations in these three genes.

A comparison of the AKT pathway-altered and unaltered groups in the CGP results is presented in Table 2. The proportion of patients with *ERBB2* amplification was lower in the AKT pathway-altered group than that in the unaltered group (4.0 vs. 5.8%, p = 0.087). The proportion of tumor BRCA1 or BRCA2 variants in tumor tissue was significantly lower in the AKT pathway-altered group (8.0 vs. 16.2%, p < 0.001). Specifically, the proportion of somatic BRCA1/2 variants, which are detected only in somatic cells and considered negative in the germline BRCA1/2 test, was significantly lower in the AKT pathway-altered group (4.7 vs 6.6%, p = 0.009).

**Table 2:** Comparison of AKT pathway-altered and -unaltered groups based on comprehensive genomic profile (CGP) results

Factor	Group	AKT pathway-altered	Unaltered	p.value
n		1038	929	
*ERBB2* amplification (%)	***ERBB2 *amplified**	**42 (4.0)**	**54 (5.8)**	**0.087**
	* tERBB2 *amplified	42 (4.0)	51 (5.5)	
	* bERBB2* amplified	0 (0.0)	3 (0.3)	
	***ERBB2* not amplified**	**996 (96.0)**	**875 (94.2)**	
Tumor *BRCA1/2* (%)	***tBRCA1* or *tBRCA2***	**84 (8.0)**	**150 (16.2)**	**<0.001**
	* tBRCA1*	14 (1.3)	22 (2.4)	
	* tBRCA2*	69 (6.6)	125 (13.5)	
	* tBRCA1 *&* tBRCA2*	1 (0.1)	3 (0.3)	
	**No variants**	**954 (91.9)**	**779 (83.9)**	
Somatic* BRCA1/2* (%)	***sBRCA1 *or* sBRCA2***	**49 (4.7)**	**61 ( 6.6)**	**0.009**
	* sBRCA1*	10 (1.0)	11 (1.2)	
	* sBRCA2*	39 (3.8)	49 (5.3)	
	* sBRCA1 *&* sBRCA2*	0 (0.0)	1 (0.1)	
	**Non* sBRCA1/2***	**989 (95.3)**	**868 (93.4)**	
MSI (%)	**MSI high**	**5 (0.5)**	**1 (0.1)**	**0.274**
	**MSI not high or not available**	**1033 (99.5)**	**928 (99.9)**	
TMB (%)	**TMB high**	**163 (15.7)**	**68 (7.3)**	**<0.001**
	(median, range)	4 (0–134.1)	3.78 (0–258.9)	
	tTMB hign	33 (3.2)	10 (1.1)	
	bTMB hign	130 (12.5)	58 (6.2)	
	(bTMB not available)	5 (0.5)	9 (1.0)	
	**TMB not high or not available**	**870 (83.8)**	**852 (91.7)**	
Tumor TMB(%)	n (total: 1524)	828	696	
	(median, range)	4 (0–134.1)	3.78（0–258.9)	
	**tTMB high**	**130 (15.7)**	**58 (8.3)**	**<0.001**
	**tTMB not high**	**698 (84.3)**	**638 (91.7)**	
Blood TMB(%)	n (total: 443)	210	233	
	(median, range)	5 (0–100)	3 (0–102)	
	**bTMB high**	**33(15.7))**	**10 (4.3)**	**<0.001**
	**bTMB not high**	**172(81.9)**	**214 (91.8)**	
	(bTMB not available)	5 (2.4)	9 (3.9)	

For each factor, the *χ*^2^ test was performed on the variables in bold

Tumor *BRCA1/2* alterations were cases in which germline *BRCA1/2* gene testing was not performed, but somatic *BRCA1/2* alterations were found; Somatic *BRCA1/2* mutations were cases in which germline *BRCA1/2* gene testing was performed and negative, but somatic *BRCA1/2* alterations were defined as cases with somatic *BRCA1/2* alterations among those with a negative germline *BRCA1/2* gene test

*tERBB2* tissue-based panel, *bERBB2* liquid-based panel, *tBRCA1* tumor *BRCA1*, *tBRCA2* tumor *BRCA2*, *TMB* tumor mutation burden, *MSI* microsatellite instability

A significantly higher proportion of TMB was observed in the AKT pathway-altered group (15.7 vs. 7.3%, p < 0.001). In detail, both tumour TMB and blood TMB were significantly higher in the AKT pathway altered group (tTMB: 15.7% vs. 8.3%, p < 0.001, bTMB: 15.7% vs. 4.3%, p < 0.001, respectively). There was no significant difference in the microsatellite instability (MSI) status between the two groups (0.3 vs. 0.3%, p = 1.000).

## Discussion

In this study, we identified various clinicopathological factors and differentially expressed genes in Japanese luminal-type mBC with and without the appearance of AKT pathway alterations. These findings emphasize the significance of developing novel treatment strategies based on the characteristics of each group. To the best of our knowledge, this is the first clinicopathological analysis of AKT pathway alterations in Japanese patients with mBC.

The frequency of AKT pathway alterations in luminal-type mBC in Japanese patients was 52.8%. And the frequency of patients with the companion diagnostic gene for capivasertib was 48.6%, which was higher than that reported in the CAPIttelo-291 study (40.8%). This may be associated with the accumulation of mutations as cancer progressed because the CAPIttelo-291 trial included early-line patients, whereas the study included late-line patients who had completed or were anticipated to complete standard treatment [15].

In our cohort, patients with mutations in the AKT pathway were older (p = 0.002), exhibited a higher proportion of histological ILC (p = 0.001), PgR positivity (p = 0.006), and bone metastases (p = 0.007), and a lower proportion of gBRCA2 (p < 0.001). In a pooled analysis of 10,319 individuals, *PIK3CA* was significantly associated with older age [16]. Additionally, *PIK3CA* mutations were associated with post-menopause and PR positivity [17]. This study demonstrated similar results, but this is the first report of ILC, BRCA2, and bone metastasis associated with AKT pathway alterations in luminal recurrent breast cancer.

The gene profile results demonstrated that the order of expression frequency was not significantly altered based on the presence or absence of the AKT pathway. However, the AKT pathway alteration group exhibited a higher proportion of TP53 expression, whereas the unaltered group exhibited a higher GATA-binding protein 3 (GATA3) co-expression (47 vs. 34%, respectively; 12 vs. 22%). In early-stage breast cancer, patients with TP53 and *PIK3CA* alterations and negative mutations after preoperative chemotherapy have enhanced disease-free and overall survival compared to those with no alterations or the opposite [18]. Data should be accumulated on the prognosis of patients with coexisting AKT pathway alterations and TP53 mutations in mBC and their association with the response to molecularly targeted therapy. Additionally, TP53 mutations have been mutually exclusive with GATA3 mutations [19], which is consistent with the present results.

Additionally, differences were observed in the presence or absence of mutations in the AKT pathway based on the factors obtained from CGP testing. *PIK3CA* mutations are common in breast cancer; however, their direct relationship with TMB remains unclear. *PIK3CA* mutant tumors with concurrent AT-rich interactive domain-containing protein 1A (*ARID1A*) mutations exhibited a higher TMB than those without *ARID1A* mutations [20]. Our study is the first to indicate that AKT pathway alterations in luminal-type mBC are associated with high TMB. The optimal clinical approach for TMB-high or-highly mutated metastatic breast cancer is unknown because of the lack of prospective data. These results suggest that some mutations, including alterations in the *PI3K/AKT/PTEN* pathway, may be enriched in TMB-high metastatic breast cancer. A possible explanation for this phenomenon is that AKT pathway alterations promote cell proliferation and survival, such that alterations are more likely to accumulate in such a selective pressure environment, and consequently, the tumor mutational burden may also remain high. The AKT pathway-targeted therapies and immune checkpoint inhibitor groups of TMB are both therapeutic options. Because there is currently no evidence supporting their combination, they should be administered sequentially. However, future studies should be accumulated to determine the optimal treatment sequence. In contrast, our results demonstrated that germline BRCA1/2 and somatic BRCA1/2 were negatively correlated with AKT pathway alterations. These results indicate that patients eligible for AKT inhibitors were less likely to be eligible for poly(ADP-ribose) polymerase (PARP) inhibitors. However, the optimal sequencing of their treatment should be assessed, and their coexistence is less frequent.

The companion genes for capivasertib include those published as FDA labels. Not all *PI3K/AKT/PTEN* variants are companion diagnostic genes. Target variants, such as *PIK3CA* amplification, are individualized based on clinical trial results and may not be covered. Capivasertib may be recommended based on test company reports and should be carefully considered when recommended by each expert panel.

This study has certain limitations: only FoundationOne CDx was validated as a companion diagnostic device for capivasertib. However, our study results are a combined analysis of both tissues and liquids based panel. It should be noted that the number of genes and analysis methods differed for each test method. In this study, the CGP results from five different panel tests were collectively analyzed, which introduced biases owing to the characteristics and types of tests. And there was insufficient prognostic data in the C-CAT database, which were not considered in this study. Accurate prognostic studies in the real world are desirable, and a breast cancer-specific registry should be established.

In conclusion, this study demonstrated that the AKT pathway alteration has a mutation rate of 52.8% in luminal-type mBC and 48.6% in the capivasertib companion diagnostic gene, which differed in clinicopathological factors. Additionally, differences were observed in the factors derived from the CGP test results. Future studies should assess how various factors affect the therapeutic effects of capivasertib.

## Supplementary Information

Below is the link to the electronic supplementary material.

Supplementary file1 (PDF 799 KB)

## Data Availability

All data are provided by the corresponding author upon request.
