# Supplementary material for: Clinicopathogenomic analysis of PI3K/AKT/PTEN-altered luminal metastatic breast cancer in Japan
Source: Breast Cancer. 2024 Oct 28;32(1):208–16. doi: 10.1007/s12282-024-01639-6 (PMC11717796; doi:10.1007/s12282-024-01639-6)
Supplement: Supplementary file 1 — Supplementary file1 (PDF 799 KB) [file 12282_2024_1639_MOESM1_ESM.pdf]

Supplementary figure 1. Flow diagram

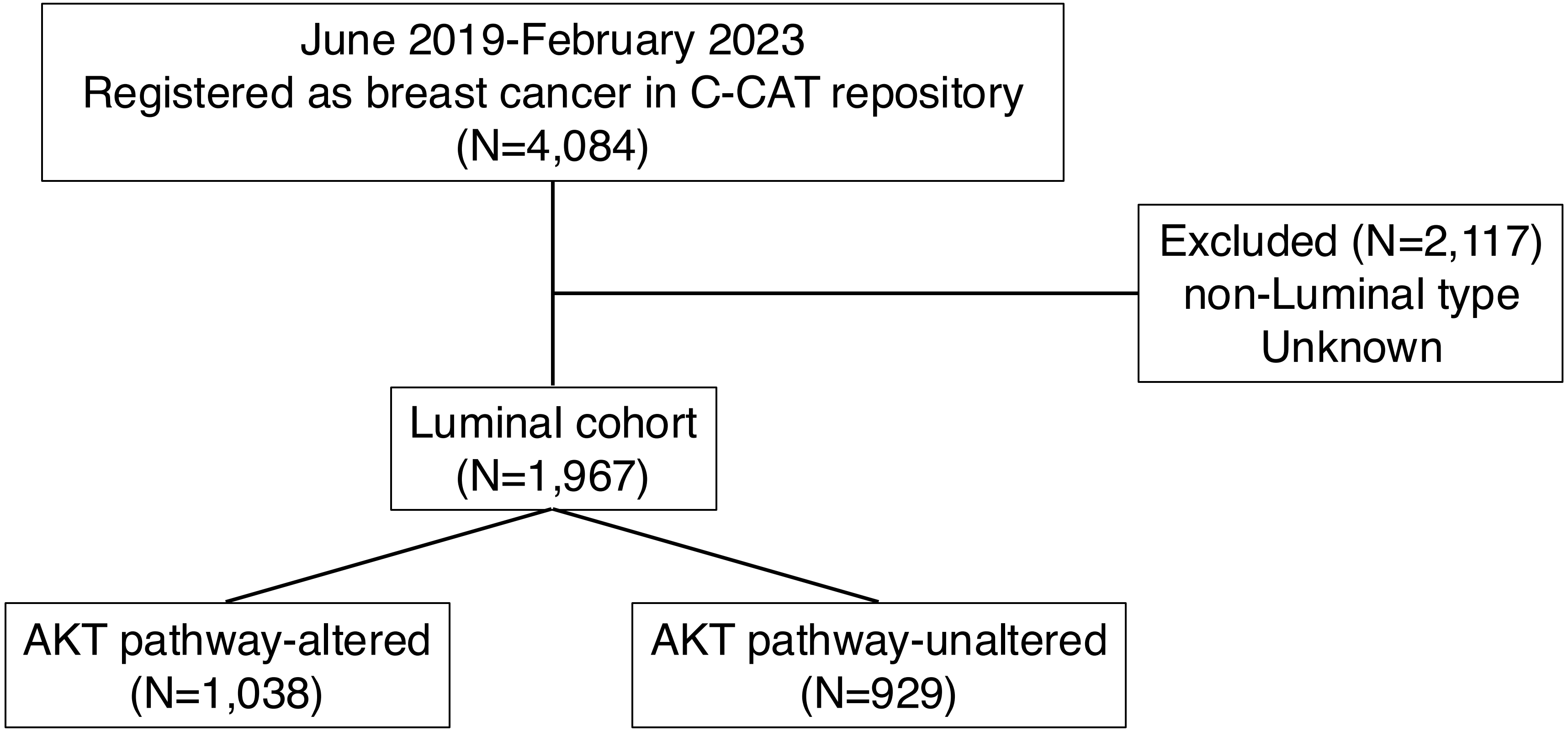

Supplementary Table 1. Frequency of AKT1, PIK3CA and PTEN alterations

Alterations (total 1364)	n
PIK3CA H1047R	323
PIK3CA E545K	168
AKT1 E17K	141
PIK3CA E542K	89
PTEN loss	66
PIK3CA H1047L	55
PIK3CA amplification	53
PIK3CA N345K	30
PIK3CA E726K	29
AKT1 amplification	16
PTEN T319fs*1	15
PIK3CA C420R	11
PTEN Q245*	10
PIK3CA G1049R	9
PIK3CA Q546K	9
PIK3CA E545A	8
PIK3CA E545G	7
PIK3CA E545Q	7
PIK3CA E970K	7
PIK3CA R88Q	7
PTEN R130Q	7
PIK3CA E418K	6
PIK3CA E81K	6
PIK3CA N1044K	6
PIK3CA E365K	5
PIK3CA E453K	5
PIK3CA G118D	5
PIK3CA M1043I	5
PIK3CA M1043V	5
PIK3CA M1004I	4
PIK3CA P539R	4
PIK3CA Q546R	4
PTEN C124S	4
PTEN D92V	4
AKT1 L52R	3
PIK3CA E542Q	3
PIK3CA N1068fs*5	3
PIK3CA V344G	3
PTEN D107Y	3
PTEN G127E	3
AKT1 D323N	2
AKT1 E17R	2
AKT1 L52H	2
AKT1 Q79K	2
PIK3CA C378F	2
PIK3CA C378R	2
PIK3CA E110del	2
PIK3CA E542V	2
PIK3CA E545D	2
PIK3CA G106_R108del	2

Alterations	n
PIK3CA G106V	2
PIK3CA H1047Y	2
PIK3CA K111E	2
PIK3CA K111N	2
PIK3CA M1043L	2
PIK3CA R93W	2
PIK3CA T1025A	2
PIK3CA T1025S	2
PIK3CA V344M	2
PTEN A126D	2
PTEN c.209+4_209+7delAGTA	2
PTEN E242fs*1	2
PTEN G132V	2
PTEN N323fs*21	2
PTEN P38fs*16	2
PTEN Q298*	2
PTEN R233*	2
PTEN T319fs*6	2
PTEN truncation	2
PTEN Y177*	2
AKT1 D323G	1
AKT1 D323H	1
PIK3CA A1066V	1
PIK3CA C604R	1
PIK3CA C901F	1
PIK3CA D1017H	1
PIK3CA D1029H	1
PIK3CA D1045N	1
PIK3CA D350G	1
PIK3CA D350N	1
PIK3CA E39K	1
PIK3CA E453D	1
PIK3CA E453Q	1
PIK3CA E542A	1
PIK3CA E600K	1
PIK3CA G1007R	1
PIK3CA G106D	1
PIK3CA G364R	1
PIK3CA G914R	1
PIK3CA H1048R	1
PIK3CA H450_P458del	1
PIK3CA I962M	1
PIK3CA N345H	1
PIK3CA N345I	1
PIK3CA N345Y	1
PIK3CA P104L	1
PIK3CA P104R	1
PIK3CA P366R	1
PIK3CA P471A	1
PIK3CA P471L	1

Alterations	n
PIK3CA Q546E	1
PIK3CA Q546P	1
PIK3CA R108H	1
PIK3CA R357Q	1
PIK3CA R93Q	1
PIK3CA S965R	1
PIK3CA T957P	1
PIK3CA Y1021C	1
PTEN 24_79+9>23	1
PTEN A126T	1
PTEN A328fs*1	1
PTEN A328fs*16	1
PTEN A79fs*21	1
PTEN c.1027-1_1027delGG	1
PTEN c.1027-1G>A	1
PTEN c.1027-2A>G	1
PTEN c.165-2A>C	1
PTEN c.165-2A>G	1
PTEN c.209+1_209+2delGT	1
PTEN c.210-2A>T	1
PTEN c.253+1G>T	1
PTEN c.253+2T>G	1
PTEN c.254-28_282del57	1
PTEN c.635-1G>C	1
PTEN c.802-1G>C	1
PTEN c.802-9_821del29	1
PTEN c.947_1026+220delinsA	1
PTEN c.982_1026+12del57	1
PTEN C124fs*10	1
PTEN C124R	1
PTEN C136fs*44	1
PTEN C136Y	1
PTEN C211*	1
PTEN C211fs*1	1
PTEN C296fs*1	1
PTEN D162fs*5	1
PTEN D297fs*10	1
PTEN D326fs*5	1
PTEN D51fs*8	1
PTEN D52fs*2	1
PTEN D92E	1
PTEN D92H	1
PTEN D92N	1
PTEN E106*	1
PTEN E288*	1
PTEN E288fs*3	1
PTEN E291fs*12	1
PTEN E314fs*3	1
PTEN E99*	1
PTEN F21fs*4	1

Alterations	n
PTEN F241fs*1	1
PTEN F241fs*11	1
PTEN F347fs*5	1
PTEN F81*	1
PTEN F90fs*9	1
PTEN G129E	1
PTEN G129R	1
PTEN G132D	1
PTEN H123D	1
PTEN H272fs*15	1
PTEN I135V	1
PTEN I28fs*16	1
PTEN I28fs*27	1
PTEN I32fs*21	1
PTEN I33del	1
PTEN K163*	1
PTEN K164fs*15	1
PTEN K183*	1
PTEN K183fs*15	1
PTEN K223fs*20	1
PTEN K237fs*18	1
PTEN K267fs*9	1
PTEN K342*	1
PTEN K342fs*1	1
PTEN K6fs*4	1
PTEN L112P	1
PTEN L146fs*34	1
PTEN L152fs*1	1
PTEN L247*	1
PTEN L295fs*12	1
PTEN L325R	1
PTEN M134fs*13	1
PTEN M134I	1
PTEN N184fs*6	1
PTEN N323fs*16	1
PTEN P103fs*11	1
PTEN P248fs*5	1
PTEN P374fs*42	1
PTEN Q171*	1
PTEN Q219*	1
PTEN Q261*	1
PTEN Q97*	1
PTEN R130*	1
PTEN R130fs*3	1
PTEN R130G	1
PTEN R130L	1
PTEN R14fs*10	1
PTEN R159G	1
PTEN R15K	1
PTEN R173C	1

Alterations	n
PTEN R47fs*7	1
PTEN S10fs*10	1
PTEN S10fs*4	1
PTEN S170I	1
PTEN S229*	1
PTEN S305fs*9	1
PTEN S59*	1
PTEN T319fs*5	1
PTEN V133fs*46	1
PTEN V158fs*22	1
PTEN V166fs*14	1
PTEN V275fs*1	1
PTEN V85F	1
PTEN Y16*	1
PTEN Y16C	1
PTEN Y174fs*1	1
PTEN Y178*	1
PTEN Y27C	1
PTEN Y315*	1
PTEN Y336*	1
PTEN Y46*	1
PTEN Y65*	1
PTEN Y68fs*6	1
PTEN Y76*	1
PTEN Y76del	1
PTEN Y88fs*1	1
